# An Exploration of the Effects of Radiofrequency Radiation Emitted by Mobile Phones and Extremely Low Frequency Radiation on Thyroid Hormones and Thyroid Gland Histopathology

**DOI:** 10.7759/cureus.17329

**Published:** 2021-08-20

**Authors:** Tasnim Alkayyali, Olive Ochuba, Kosha Srivastava, Jasmine K Sandhu, Christine Joseph, Sheila W Ruo, Ashish Jain, Ahsan Waqar, Sujan Poudel

**Affiliations:** 1 Pathology, California Institute of Behavioral Neurosciences & Psychology, Fairfield, USA; 2 Internal Medicine, California Institute of Behavioral Neurosciences & Psychology, Fairfield, USA; 3 Neurology, California Institute of Behavioral Neurosciences & Psychology, Fairfield, USA; 4 Family Medicine, California Institute of Behavioral Neurosciences & Psychology, Fairfield, USA; 5 General Surgery, California Institute of Behavioral Neurosciences & Psychology, Fairfield, USA

**Keywords:** mobile phones, cell phones, electromagnetic field, radiofrequency, microwaves, low-frequency radiation, thyroid hormones, thyroid gland, thyroid cancer

## Abstract

The use of mobile phones has widely increased over the last two decades. Mobile phones produce a radiofrequency electromagnetic field (RF-EMF), a form of non-ionizing radiation. In contrast to the ionizing radiation proven to cause DNA damage, the harmful effects of non-ionizing radiation on the human body have not been discovered yet. The thyroid gland is among the most susceptible organs to mobile phone radiation due to its location in the anterior neck. Our purpose in this literature review is to explore the effects of the electromagnetic field (EMF), especially radiofrequency emitted from mobile phones, on thyroid hormones and thyroid gland histopathology. We searched PubMed and Google Scholar databases for relevant studies published after the year 2000, using the following keywords: ‘cell phones', ‘mobile phones', ‘telephones', ‘electromagnetic fields', ‘radiofrequency radiation', ‘microwaves', ‘thyroid gland', ‘thyroid hormones', and ‘thyroid cancer'. Our review revealed that mobile phone radiofrequency radiation (RFR) might be associated with thyroid gland insufficiency and alterations in serum thyroid hormone levels, with a possible disruption in the hypothalamic-pituitary-thyroid axis. The review also showed histopathological changes in the thyroid gland follicles after exposure of rats to non-ionizing radiation. The results were directly related to the amount and duration of exposure to EMF radiation. Further human studies exploring thyroid gland hormones, microscopic morphology, and thyroid cancer are highly recommended for future researches.

## Introduction and background

'I do not doubt in my mind that, at present, the greatest polluting element in the earth’s environment is the proliferation of electromagnetic fields (EMFs),' said Dr. Robert O. Becker (1923 − 2008), a researcher from the United States (US) in electromedicine and a Nobel Prize winner [1]. In 2021, mobile phone subscriptions surpassed eight billion users worldwide, and the number is expected to increase to 8.8 billion by 2026 [2]. Mobile phones use EMFs with frequencies ranging from 450-3800 MHz [3]. The EMF comprises both an electric field and a magnetic field; the electric field is produced between positive and negative electric charges and, in contrast, a magnetic field can be generated by the movement of electrons, known as electric current [4]. Electromagnetic waves are classified based on their frequencies, i.e., the number of cycles per second, measured in Hertz (Hz) [4]. High-frequency EMFs (HF-EMFs), including gamma rays, X-rays, and higher ultraviolet lights, are forms of ionizing radiation, therefore, capable of breaking the DNA bonds of human cells [5].

**Figure 1:**
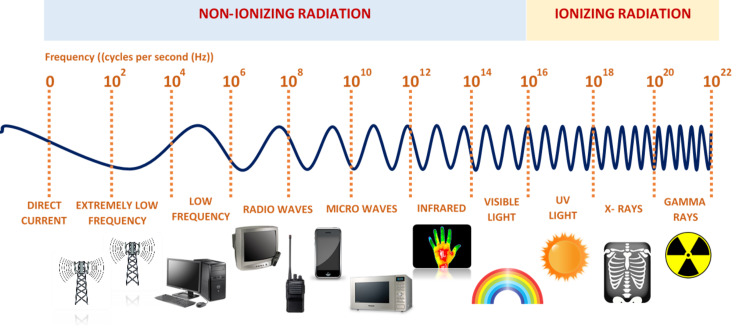
The electromagnetic spectrum with ionizing radiation having the highest radiation frequencies Modified from Diab et al. [4].

Non-ionizing forms of radiation include lower frequencies on the electromagnetic spectrum and are not proven to cause DNA damage directly (Figure 1 ). Examples of extremely low frequency-EMFs (ELF-EMF) include electricity from power sockets at homes, power lines, and electrical devices such as hair dryers [5]. Radiofrequency-EMF (RF-EMF) is also a subtype of non-ionizing radiation with frequencies ranging from 30 kHz-300 GHz [6]. Radiofrequency-based technology has increased dramatically over the last few decades; it includes mobile phones, computer monitors, tablets, radio and television broadcasting antenna towers, wireless fidelity (Wi-Fi), radars, MRI, and microwave ovens [6].

Several pieces of research were conducted to study the health effects of ELF-EMF and RF-EMFs. One of the most pronounced effects found was the heating of human tissues, i.e., the thermal effect [7]. When exposed to EMF, some of the radiation is absorbed by our bodies, while some are reflected away. The absorbed energy is measured by 'specific energy absorption rate' (SAR), which is the power absorbed per unit mass (Watt/kg) [7]. Inside a human body, a portion of that energy is converted into kinetic energy, causing friction and heat production. The body can accommodate small increases in its temperature; however, above a specific limit (threshold), depending on the exposure duration, serious health effects can occur to human cells such as that caused by burns or heat strokes [7]. Therefore, radiofrequency threshold guidelines were developed by the International Commission on Non-Ionizing Radiation Protection (ICNIRP) [7] as well as by the Institute of Electrical and Electronics Engineers (IEEE) [8]. These thresholds are intended to guide manufacturers of electric devices, including cell phones, on the maximum radiation limits allowed to be emitted by those devices. In addition to thermal effects, EMF can affect the body by inducing an electrical current that can stimulate nerves inside the body and change the permeability of cell membranes [7]. The changes in cell metabolism without affecting its temperature are referred to as the non-thermal effects of EMF radiation [9].

Some health-related concerns have increased along with the revolution of mobile phone usage. These include male infertility, cognitive and memory deficits, headaches, sleep problems, and increased cancer risk [10]. According to research, lower intensity EMF may lead to cellular stress, increased blood-brain barrier permeability, reactive oxygen species formation, and genetic damage [11]. The INTERPHONE international study found that frequent cell phone users have a possible increased risk of developing brain tumors in the temporal lobe, especially glioma and meningioma [12]. Therefore, the International Agency of Research on Cancer (IARC) classified ELF-EMF and RF-EMR as Group 2B 'Possible Carcinogen' [6]. Although the relationship to cancer is not yet well established, EMF exposure over the long term might lead to harmful effects in the human body; the brain and thyroid gland are among the most susceptible organs due to their proximity to mobile phone radiation [4].

The purpose of this review is to explore the effects of EMF, especially radiofrequency radiation (RFR) emitted from mobile phones, on the thyroid gland hormones and thyroid tissue. We aimed to look at alterations in thyroid hormone levels upon exposure of the thyroid gland to non-ionizing radiation for a specific duration. Additionally, we reviewed the histopathological effects of non-ionizing radiation on the thyroid gland follicular tissue. We also wanted to determine whether RF-EMF or ELF-EMF is related to increased thyroid insufficiency in recent years. Therefore, we searched human, animal, and in-vitro studies in PubMed and Google Scholar databases and included studies done after the year 2000 using the following keywords: ‘cell phones', ‘mobile phones', ‘telephones', ‘electromagnetic fields', ‘radiofrequency radiation', ‘microwaves', ‘thyroid gland', ‘thyroid hormones', and ‘thyroid cancer'. We retrieved a total of 138 articles and included only the articles that directly tested the association between non-ionizing radiation exposure and the thyroid gland. We ended up with 28 articles that were most relevant to our topic. Of these articles, six were observational human studies, 18 were animal studies, three were in-vitro studies, and one was a systematic review of mixed studies.

## Review

Mobile phones and thyroid gland hormones

Thyroid gland hormones, including triiodothyronine (T3) and thyroxine (T4), are essential hormones for regulating the basal metabolic rate (BMR) of the body. Of the thyroid gland secretions, 90% are T4 that later converts into T3, the more potent and active form of thyroid hormones [3]. In addition, the thyroid-stimulating hormone (TSH) regulates the serum levels of these thyroid hormones. The anterior pituitary releases TSH which then acts on the TSH receptors present on the thyroid gland follicles [3]. In the systematic analysis done by Asl et al. (2019), two studies reported decreased TSH levels in subjects exposed to radiation, while one reported an increase [3]. The systematic review also included five studies with decreased T4 levels upon radiation exposure and two studies that reported an increase [3]. Additionally, six studies revealed a decreased T3 levels, while one showed an increase [3]. The systematic review explained that exposure to microwaves could cause cellular stress and increased thyroid gland temperature; this can negatively affect the iodine uptake by the thyroid follicles, leading to thyroid dysfunction [3].

Thyroid Hormones in Human Studies

Several studies investigated the effects of RFR emitted by global systems for mobile communications (GSM) mobile phones on serum thyroid hormone levels. In a medical college in South India, Baby et al. (2017) performed a cross-sectional study on 83 undergraduate students [9]. Surveys questioned the types of cell phones used and their SAR values, along with the duration of usage. The results showed a significant association between the total amount of radiation exposure and increased serum TSH levels, indicating a possible hypothyroid state in excessive mobile phone users [9]. Another cohort study was done by Eskander et al. (2012) using 82 volunteers divided into groups based on their RFR exposure duration and intensity [13]. The source of RFR exposure was 950 MHz emitted from mobile phones. After a periodic follow-up for six years, blood results showed that those who had high and prolonged exposure to RFR suffered a significant (P<0.01) decrease in their T3 and T4 levels, indicating a possible thyroid dysfunction [13]. Mortavazi et al. (2009) performed a cross-sectional study on 77 healthy university students. The results showed high TSH, low T4, and normal T3 concentrations in excessive mobile phone users, indicating a possible hypothyroid state and thyroid dysfunction directly proportional to the degree of RFR exposure duration and intensity [14]. The study also concluded a possible harmful effect of cell phone RFR on the hypothalamic-pituitary-thyroid (HPT) axis [14].

Bergamaschi et al. (2004) performed a cross-sectional study to analyze the effects of cell phones on the thyroid gland function of 2598 employees [15]. Bergamaschi et al. divided the workers into groups based on their cell phone usage frequency, using a questionnaire. The group of employees who had conversations on their mobile phones for > 33 hours per month had a 9.9% prevalence of low TSH values compared to a prevalence of 6% in the group who talked on their mobile phones for <19 hours per month (P<0.05) [15]. Based on the above findings, mobile phone radiation could negatively affect thyroid gland function and hormone production directly by disrupting the thyroid gland tissue or indirectly by disrupting the HPT axis. For that reason, we can observe either a decrease or an increase in serum TSH values together with thyroid insufficiency upon RFR exposure.

Thyroid Hormones in Animal Studies

Many animal studies sought to discover the association between non-ionizing radiation and thyroid hormones. Izmest'eva et al. (2003) performed an experimental animal study by exposing laboratory rats to 12 minutes of microwave radiation using a SAR value of 30W/Kg, a very high absorption rate [16]. The study concluded that non-ionizing radiation could lead to a transient disruption of the hypophysis-thyroid system, causing a hypothyroid state in rats [16]. The study also noted that long-term exposure to microwaves could cause thyroid insufficiency and primary thyroiditis in animal rats [16]. Pawlak et al. (2014) explored the blood plasma of chick embryos and newly hatched chicks after being exposed to 1800 MHz radiation for 12 and 18 days. As a result, serum T4 and T3 concentrations were significantly decreased in the embryos and the newly hatched chicks [17]. This study determined that exposure to 1800 MHz radiofrequency EMF can inhibit the function of the HPT axis in animals and lead to a hypothyroid state during embryogenesis [17]. Moreover, Hajioun et al. (2014) aimed to test the deleterious effects of mobile phone radiation together with the protective effects of garlic on the pituitary-thyroid hormones [18]. The study divided laboratory rats into groups, including a control group and a group exposed to 900 MHz. T4 and T3 hormones decreased one month later, but TSH increased significantly in all groups compared to controls, indicating a hypothyroid state [18]. Moreover, garlic consumption did not show a significant reduction in the adverse effects of cell phones on the thyroid gland [18]. 

In the study of Koyu et al. (2005), 30 rats were used and divided into groups. The exposure group was exposed to 900 MHz EMF for 30min per day for one month [19]. Results showed that serum TSH, T3, and T4 values in the exposure group were all significantly lower than those in the sham-exposed group (p<0.01) [19]. Another animal study was done by Peighambarzadeh et al. (2017), in which 60 rats were exposed to cell phone RFR between 500 to 900 MHz for 21 days [20]. As a result, mean TSH levels decreased in the test group compared to the control group, indicating a disrupted HPT axis [20]. Mohammadi et al. (2015) also found an inhibitory effect on the thyroid hormones when rats got exposed to 940 MHz mobile phone radiation for one, three, and six hours per day for two months [21]. Serum T3 concentrations decreased significantly in all groups compared with the control group (p<0.001) [21]. Serum T4 concentrations decreased significantly in the groups exposed to cell phone radiation for three and six hours per day only (not in the group exposed for one hour per day), indicating an inhibitory effect on the thyroid hormones proportional to EMF exposure duration [21].

The majority of studies point towards an inhibitory effect of RFR on the HPT axis or thyroid tissue, leading to thyroid insufficiency, especially over the long term. In contrast, few studies did not find significant results. Kim et al. (2013) used a whole-body exposure system with a reverberation chamber to emit Radiofrequency Identification (RFID) on laboratory rats [22]. However, this study found no statistically significant T3, T4, or TSH changes after exposure to 915 MHz RFID [22]. The in-vitro studies performed by Asl et al. (2013) and Mahmoudi et al. (2014) used serum sample cells of healthy donors and exposed the samples to 900MHz from a GSM mobile phone simulator for 18 and 30 minutes, respectively [23,24]. The studies found no statistically significant differences in serum T3 levels of the exposed groups of cells compared to controls (P > 0.05) [23,24]. The differences in findings might be due to the differences in study types, duration of radiation exposure, the intensity of radiation, and the technological equipment used. These in-vitro studies used a short duration of exposure with a small SAR value of 1.09 W/kg compared to the SAR value of 30W/Kg used in the study of Izmest'eva et al. [16]. The insignificant results also support the hypothesis that the harmful effect of non-ionizing radiation is directly proportional to the duration and intensity of exposure. Table 1 summarizes the above-mentioned studies.

**Table 1:** Non-Ionizing radiation and thyroid gland hormones EMF: Electromagnetic field; TSH: Thyroid stimulating hormone; T3: Triiodothyronine; T4: Thyroxine; NA: Not applicable; SAR: Specific energy absorption rate; HPT: Hypothalamic-pituitary-thyroid; ↓: Decreased; ↑: Increased; m: Meter; MHz: Megahertz; W/kg: Watt per kilogram

Author (Year)	Study design	Species studied & number of subjects	Variable studied	EMF exposure duration & intensity	Study results
Asl et al. (2019) [3]	Systematic Review	22 studies; (comprising 7182 cases)	T3, T4, & TSH	NA	↓T3 in six studies and ↑ T3 in one study. ↓T4 in five studies and ↑ T4 in two studies. ↓ TSH in two studies, and ↑ TSH in one study.
Baby NM et al. (2017) [9]	Cross-Sectional	83 undergraduate students	TSH	NA	↑ TSH correlated with ↑ the total amount of cell phone exposure.
Eskander et al. (2011) [13]	Cohort	82 human volunteers (followed for six years)	T3 & T4	950 MHz mobile phones or living at a distance of 20-100m & 100-500m away from a base station.	↓ T3 and T4 in the exposure group.
Mortavazi et al. (2009) [14]	Cross-Sectional	77 healthy university students	T3,T4, & TSH	Mobile phone usage duration.	↑ TSH, ↓ T4, and normal T3 in excessive mobile phone users. Possible disruption of the HPT axis.
Bergamaschi et al. (2004) [15]	Cross-Sectional	2598 human employees	TSH	900MHz from mobile phones with conversation time >33hours/month.	↓TSH among workers with > 33 hours/month conversation time.
Izmest'eva et al. (2003) [16]	Animal	Laboratory rats	HPT axis	Microwaves with SAR of 30 W/kg for 12-minutes.	Thyroid gland insufficiency (primary hypothyroidism) was observed in the exposed rats.
Pawlak et al. (2014) [17]	Animal	Chick embryos, newly hatched chicks, and birds ready for slaughter	T3, T4	1800 MHz, for four min, 10 times per day, for 12 and 18 days.	↓ T3 and T4 in the embryos and the newly hatched chicks but not in birds ready for slaughter. Inhibition of the HPT axis.
Hajioun et al. (2014) [18]	Animal	40 Wistar rats	T3, T4, & TSH	900 MHz emitted from Nokia 1200 cell phone 12 times a day, each time 10 minutes for one month.	↓ T3 and T4, and ↑ TSH in the exposure group.
Koyu et al. (2005) [19]	Animal	30 adult male Sprague-Dawley rats	T3, T4, & TSH	900 MHz for 30 minutes per day, five days per week for one month.	↓ T3 and T4, and ↓TSH in the exposure group.
Peighambarzadeh et al (2017) [20]	Animal	60 adult Swiss albino mice	TSH	500 to 900 MHz twice a day for 21 days.	↓TSH in the exposure group.
Mohammadi et al. (2015) [21]	Animal	Male Wistar rats	T3 & T4	940 MHz for one, three, and six hours/day for two months.	↓T3 in those exposed to radiation for one, three, and six hours/day, and ↓ T4 in those exposed for three and six hours/day only. Thus, the inhibitory effects on thyroid hormones secretion are proportionally related to exposure duration.
Kim et al. (2013) [22]	Animal	162 male Sprague–Dawley rats	T3, T4, & TSH	915 MHz; SAR 3.2-4.6. For eight hours /day, five days per week, for 16 weeks.	No significant changes in T3, T4, or TSH between the exposure and control groups.
Asl et al. (2013) [23]	In Vitro; Human Serum	29 healthy donors	T3	900MHz from mobile phones for 18 minutes.	No significant difference in serum T3 between the exposure and control groups.
Mahmoudi et al. (2014) [24]	In Vitro; Human Serum	63 healthy donors	T3	900 MHz from a mobile phone (Nokia, Model 1202, India); SAR: 1.09 W/kg, for 30 minutes.	No significant difference in T3 between the exposure and control groups.

Mobile phones and thyroid gland histopathology

The thyroid gland is composed of follicles, which are spherical structures lined by cuboidal cells, i.e., follicular cells, and are responsible for thyroid hormone production. In the middle of a follicle, there is colloidal material composed of thyroglobulin [3]. In addition, parafollicular cells are present in between thyroid follicles and secrete calcitonin, which is involved in calcium homeostasis [3]. Many studies in the literature studied the histopathological and morphological effects of non-ionizing radiation on the thyroid gland. In the systematic review done by Asl et al. (2019), seven studies showed a reduced volume and diameter of follicular cells in the thyroid glands exposed to microwaves [3].

Thyroid Gland Histopathological Changes Due to 900 MHz EMF

Many animal studies explored the effects of RFR at the microscopic level of the thyroid gland tissue. Eşmekaya et al. (2010) exposed animal rats to 900MHz for three weeks and observed the histopathologic changes in the thyroid gland. The study found significant pathological changes consistent with hypothyroidism [25]. There was a significant decrease in follicular epithelial height and an increase in the area and diameter of the colloid; both findings indicate a resting state of follicular cells [25]. When the activity of thyroid follicles decreases, phagocytosis of colloidal material also decreases, leading to inhibition of thyroid hormones synthesis and secretion. Thus, the colloidal material accumulates inside the follicular lumen and minimizes the height of the surrounding follicular cells. The lower the height of follicular epithelium, the lower its activity [25]. Moreover, electron microscopy revealed an increase in apoptotic bodies in the exposure group; caspase-3 (the effector) and caspase-9 (the initiator)-dependent apoptotic pathways were activated [25]. This increase in apoptosis indicates a cellular stress effect induced by 900 MHz radiation on the thyroid gland follicles.

The histopathologic changes induced by RFR are not limited to a resting follicular state. Most animal studies revealed hyperstimulation of the thyroid follicles. Shaukat et al. (2011, 2013) exposed animal rats to 50 missed calls per day emitted from 900/1800 MHz GSM mobile phones over two months. The study found histopathological changes in the thyroid gland consistent with gland hyperstimulation [26,27]. The experimental group’s thyroid glands revealed an abundance of micro follicles with minimal colloid material, and the mean diameter of the thyroid follicles decreased significantly (P<0.05) in the exposure group compared to controls [26,27]. Additionally, there was a significant increase in the mean height of follicular epithelial cells of 7.26± 0.24μm in the exposure group in comparison to the height of 3.60 ± 0.12μm in the control group. Also, there was an increase in the connective tissue thickness and blood capillary size in the exposed rats [26,27]. All these findings indicate hyperstimulation of the thyroid gland upon exposure to RFR; smaller follicular sizes indicate that the synthesis and secretion of thyroid hormones occur rapidly to meet the increased demands of the stimulated thyroid gland [26,27]. Hajioun et al. (2014) exposed rats to 900 MHz for one month and observed the histopathological changes in the thyroid gland. There was a reduced number of cuboidal cells, in addition to a reduced follicular colloidal fluid and follicular diameter [18]. Despite the histopathological findings of hyperstimulated thyroid follicles, T3 and T4 levels were low in the serum [18]. These findings indicate a disruption of the proper functioning of the thyroid follicular cells by RFR, leading to apoptosis and cell number reduction.

Cellular Stress Induced by EMF

Heat shock proteins (HSPs) are cellular chaperones that regulate cellular protein folding in response to external stressors or increased cellular heat. Their presence is crucial for preventing various diseases and cancerous changes. Agustiño et al. (2019) found that HSP-90 and HSP-70 significantly decreased (P<0.01) after 90 minutes of exposure to 2.45GHz [28]. However, 24 hours after stopping radiation exposure, HSP-90 levels partially returned to normal, and HSP-70 recovered completely [28]. These results suggest that EMF radiation may cause an initial reversible cellular stress but can hypothetically transform to permanent stress injury if the exposure duration was prolonged. Martín et al. (2021) also studied the effect of 2.45 GHz RFR on parafollicular cells using 24 animal rats [29]. After 90 minutes of radiation exposure, calcitonin-positive parafollicular cells significantly increased in number and size in the thyroid tissue of the exposed rats [29]. Additionally, HSP-90 inside parafollicular cells significantly decreased and remained low until 24 hours after stopping the radiation [29]. These findings suggest that RFR acts as a negative external stress stimulus and leads to parafollicular cell homeostasis dysfunction. These stress injuries might play a role in thyroid gland disorders.

Hussien et al. (2020) decided to expose 30 rats to cell phone radiation for 30 days and measure thyroid tissue oxidative stress, thyroid functions, and plasma nesfatin-1 levels [30]. Immunohistochemical and histological examination showed increased oxidative stress markers and cellular apoptosis markers in the thyroid glands after radiation exposure. Also, there was a statistically significant relationship between serum nesfatin-1 levels and oxidative stress markers, apoptosis markers, and thyroid function markers [30]. Therefore, serum nesfatin-1 levels also have a role in thyroid insufficiency caused by RFR exposure.

Studies observed that mobile phones can exert their deleterious effects on the thyroid gland through thermal and non-thermal effects by exciting cellular receptors and causing disruption in the microtubules between cells [26]. The non-thermal effect of radiation leads to reactive oxygen species (ROS) formation and accumulation of heavy metals within cells [26, 27]. RFR may stimulate NADPH oxidase present on the plasma membrane of cells, leading to ROS formation and apoptosis induction [25]. This oxidative stress may also disrupt the cellular Ca2+ ion pumps, transporters, and binding proteins [25]. In addition, RFR itself may act on Ca2+ pumps leading to Ca2+ efflux from cells and cellular apoptosis [25]. Other cellular stressors include increased cortisol levels and enzymes involved in thyroid homeostases, such as anti-thyroperoxidase, liver deiodinase, and ornithine carboxylase [18].

Thyroid Gland Histopathological Changes Due to 50Hz ELF-EMF

The effects of extremely low-frequency EMF (ELF-EMF) on thyroid gland histology were also studied in the literature. Rajkovic et al. (2001) observed the thyroid glands of animal rats under light and electron microscopes upon exposure to 50 Hz ELF-EMF [31]. After three months of exposure, there was a decrease in the thickness of the follicular cuboidal epithelium and interfollicular connective tissue [31]. In addition, both T4 and T3 serum levels decreased in the exposed rats compared to the controls [31]. These findings indicate a generally reduced thyroid gland activity upon exposure to ELF-EMF for three months. The results were slightly different when Rajkovic et al. (2006) did a similar experiment with a shorter duration of exposure to ELF-EMF. After one month of exposure to 50Hz, hyperplasia and hypertrophy developed in follicular cells, interfollicular connective tissue, and blood capillaries [32]. The study observed micro follicular arrangement of thyroid lobules containing low colloid material in the exposed group of rats in contrast to the colloid-rich macro follicles found in the control group [32]. These findings suggested an increased thyroid gland activity after one month of exposure to ELF-EMF. Therefore, the study indicated that the degree of morphological changes in the thyroid gland is directly related to the duration of exposure to ELF-EMF. There was a resting state in the thyroid follicles when exposed to RFR for three months, compared to an active state of thyroid follicles when exposed to RFR for one month. However, both states led to thyroid hormone insufficiency in the serum [31, 32].

Another study concluded that EMF radiation acts as a stress factor on the degranulation of mast cells [33], as there was an increased number of type A degranulated mast cells in the thyroid glands of rats exposed to 50 Hz EMF compared to controls [33,34]. The dilated blood capillaries observed in the exposure groups might be due to the effect of mediators released from these mast cells situated around the thyroid follicles and vasculature [33]. Rajkovic et al. (2005c) further studied the influence of ELF-EMF on mast cells, parafollicular cells, and nerve fibers in the thyroid gland of male rats [35]. Immunohistochemical analysis showed a significant increase in Neuropeptide-Y (NPY) nerve fibers and an increase in histamine-containing mast cells in the thyroid glands of the exposed rats [35]. NPY and histamine are amino-peptides that act as vasoconstrictors of the microvasculature, leading to increased blood flow and capillary permeability in the thyroid gland; thus, enhancing the uptake of substrates and stimulating the thyroid follicular cells [35]. Theoretically, this also enables more TSH to be driven via the bloodstream into the thyroid gland and have a stimulatory effect on the gland [33]. Although the structural changes in the thyroid gland due to 50Hz EMF exposure were not severe enough to damage the thyroid tissue, these changes are still significant, and the effects of higher frequencies required more investigations [32]. Table 2 summarizes the above-mentioned studies.

**Table 2:** Non-ionizing radiation and thyroid gland histopathology EMF: Electromagnetic field; MHz: Megahertz; GHz: Gigahertz; NA: Not Applicable; SAR: Specific energy absorption rate; HSP: Heat shock protein; micro T: MicroTesla; NPY: Neuropeptide-Y; ELF: Extremely-low frequency; ↓: Decreased, ↑: Increased, W/kg: Watt per kilogram

Author (Year)	Study design	Species studies & number of subjects	EMF exposure duration & intensity	Study results
Asl et al. (2019) [3]	Systematic Review	22 studies (comprising 7182 cases)	NA	↓ Volume of thyroid gland follicles and cells in seven studies.
Eşmekaya et al. (2010) [25]	Animal	30 male Wistar Rats	900 MHz at SAR of 1.35 W/Kg. For 20 minutes/day, for three weeks.	Hypothyroidism morphology of thyroid follicles. ↑ caspase-3, caspase-9, and apoptotic bodies.
Shaukat et al. (2011) [26]	Animal	20 adult Bal/b male mice	900/1800 MHz mobile phone. 50 missed calls /day, each call lasting 30 seconds. For two months.	↑ Mean height of follicular epithelial cells of the exposure group, ↓colloid. Hyperstimulation of the thyroid gland.
Shaukat et al. (2013) [27]	Animal	20 adult Bal/b male mice	900/1800 MHz mobile phone. One hour /day. For two months.	↓ Follicular size in the exposure group. Hyperstimulation of the thyroid gland.
Hajioun et al. (2014) [18]	Animal	40 Wistar rats	900 MHz. 12 times/ day, each time 10 minutes, for one month.	↓ Number of cuboidal cells, amount of follicular fluid, and follicular diameter in the exposure group. Hyperstimulation of the thyroid gland.
Agustiño et al. (2012) [28]	Animal	54 adult female Sprague-Dawley Rats.	2.45GHz exposure for 90 minutes with SAR of 0.046±1.10 W/Kg or 0.104±5.10W/Kg.	↓ HSP-90 and HSP-70. 24 hours after radiation, HSP-90 partially returned to normal, and HSP-70 recovered completely.
Martín et al. (2021) [29]	Animal	42 Sprague Dawley rats	2.45 GHz exposure for 90 minutes	↑ Calcitonin-positive parafollicular cells in the exposure group. ↓ HSP-90 in parafollicular cells with 90 minutes of radiation and remained low until 24 hours after radiation.
Hussien et al. (2020) [30]	Animal	30 adult male rats	Nokia N70 mobile phones. For 30 days. One and two hours per day.	↑ Oxidative stress and cellular apoptosis in the thyroid glands of the exposure group. There is a significant association between serum nesfin-1 levels and oxidative stress markers, apoptosis markers, and thyroid function markers.
Rajković et al. (2001) [31]	Animal	Mill Hill male rats	50 Hz (with intensity 500 microT to 50 microT, 10 V/m field). For seven hours/day, five days /week, for three months.	↑ Volume density of thyroid follicles, parafollicular cells, and mast cells. Also, ↓ thickness of the cuboidal epithelium, intrafollicular colloid, and interfollicular connective tissue. Reduced activity of the gland.
Rajkovic et al. (2006) [32]	Animal	30 Wistar male rats	50 Hz EMF (with intensity 100-300 microT, 54-160 V/m). For four hours/day, five days/week for one month	Hyperplasia and hypertrophy of follicular cells, interfollicular connective tissue, and blood capillaries. Micro follicular arrangement of thyroid lobules minimal colloid material. Increased activity of the gland.
Rajkovic et al (2005b) [33]	Animal	24 Wistar Male rats	50 Hz (with intensity 100-300 microT, 54-160 V/m). For four hours/day, seven days/week, for one month	Type A mast cells significantly increased in the thyroid of the exposure group compared to controls.
Rajkovic et al (2005a) [34]	Animal	89 Mill Hill male rats	50 Hz (with intensity 500 microT to 50 microT, 10 V/m). For seven hours/day, five days/week, for three months.	↑ Volume density of degranulated mast cells and morphological abnormalities.
Rajkovic et al (2005c) [35]	Animal	24 Wistar male rats	50 Hz (with intensity 100-300 microT, 54-160 V/m). For four hours/ day, seven days/week, for one month.	↑ NPY- nerve fibers in the thyroid glands of rats exposed to ELF-EMF compared to controls. A possible effect on thyroid vasculature.

Are mobile phones related to increased trends of thyroid gland disorders and thyroid cancer?

Studies about the relationship between RFR and thyroid cancer are lacking, and future research is required to find any association. Carlberg et al. (2020) reviewed the Swedish Cancer Register to study the thyroid cancer trends between 1970 and 2017 in the Nordic countries [36]. The study found a statistically significant increase in the average annual percentage change (AAPC) of +2.13% in the incidence of thyroid cancer in women, with a 0.95 CI (1.43- 2.83). Furthermore, the increased incidence of thyroid cancer was prominent between 2010-2017, with AAPC +9.65% and 0.95 CI (6.68-12.71) [36]. This study raised the argument that the increased exposure to RFR emitted from mobile phones in the last two decades could be a causative factor for the increased incidence of thyroid cancer, as this increase cannot be attributed to improved diagnostic measures alone [36]. Luo et al. (2020) investigated 823 single nucleotide polymorphisms (SNPs) in the human genome and revealed that 10 SNPs were significantly (P<0.01) associated with increased thyroid cancer risk in cell phone users [37]. The study discovered that genetic susceptibility might modify the association between mobile phone use and future thyroid cancer risk [37].

Children under 16 years of age are more prone to the long-term harmful effects of radiation exposure [38]. Lu & Mu (2016) conducted an experimental study using a 3D child and a 3D adult head-neck models and exposed them to 1750 MHz [38]. The study revealed that the SAR value absorbed by the child model was significantly higher than that of the adult model, indicating that children are at higher risk of radiation. It also concluded that long-term exposure to mobile phones with high SAR values might be a risk factor for various thyroid disorders in both children and adults [38].

Limitations

Most of the studies in this review conducted their experiments on animal rats in laboratory settings, using specific radiation frequencies to mimic mobile phone radiation on the thyroid glands of animals. Studying the effects of radiation on the thyroid glands of humans and taking human thyroid biopsies would require invasive procedures. In addition, conducting clinical trials on humans would require ethical considerations. Moreover, the mobile phone industry is an emerging research area with only limited data available in databases. Mobile phones can be viewed as cigarettes when they first emerged into the market; it took researchers several decades to determine the association between cigarette smoke and cancer. Thus, time might be the only determinant of an association between prolonged exposure to non-ionizing radiation and thyroid diseases or cancers in humans.

Recommendations for mobile phone users

During outgoing calls, mobile phones emit much more radiation than incoming calls [39]. Therefore, we advise not to hold the phone close to the head when connecting to other subscribers, to use a handset Bluetooth device when calling, to minimize the number and duration of calls to 3-5 minutes, to alternate the position of the phone between the right and left sides of the head, and to use airplane mode while sleeping [39]. We also recommend keeping the phone 30-40 cm away from the body when calling, sending messages, and accessing the internet [40]. Finally, when buying a phone, make sure to check the SAR value, which is a measure of the human body's absorption of EMF [39]. A SAR value of 1.6 W/kg is the limit set by the Federal Communications Commission (FCC) for public exposure to cell phones [41]. For example, the head SAR value for iPhone 11, iPhone 12, and Samsung Galaxy A71 is 1.09 W/kg, 0.99 W/kg, and 0.983, respectively [42]. Although these cellular phones follow the SAR limit, users may try to limit their duration of exposure during cellular hotspot usage and outgoing calls, as these actions have much greater SAR values [41]. With the rising commercial mobile phone industry, new cellular devices may exceed the SAR limit set by the FCC, which requires further attention. 

## Conclusions

This article aimed to explore the effects of RF-EMF and ELF-EMF on the thyroid gland hormones and histopathology. Studies collected in this review showed that GSM mobile phone RFR could be associated with alterations in T3, T4, and TSH serum hormone levels. EMF emitted from mobile phones could disrupt the function of the HPT axis and lead to thyroid insufficiency. In addition, EMF could lead to hyperstimulation of thyroid gland follicles, causing oxidative stress and apoptosis of follicular cells. Most studies revealed a proportional correlation between thyroid gland dysfunction and the exposure duration, intensity, and SAR value of radiation. Moreover, non-ionizing radiation was seen to be significantly associated with histopathological changes in the thyroid gland follicles. The exposure duration and intensity also determined the degree of morphological damage occurring in the thyroid gland tissue. Non-ionizing EMF radiation might be responsible for the recent increase in the incidence of thyroid insufficiency and cancer in the general population. However, not enough data was found related to thyroid cancer risk with non-ionizing radiation exposure. Keeping in mind the ethical considerations, we recommend future observational studies be conducted on human beings to further explore the association of non-ionizing radiation emitted from mobile phones on the thyroid gland's hormones, histopathology, and cancers over the long term.
